# Association between four microRNA binding site-related polymorphisms and the risk of warfarin-induced bleeding complications

**DOI:** 10.17179/excli2019-1352

**Published:** 2019-05-28

**Authors:** Maryam Hosseindokht, Mohammadali Boroumand, Rasoul Salehi, Ali Mandegary, Azita Hajhosseini Talasaz, Leyla Pourgholi, Hamed Zare, Shayan Ziaee, Mohammadreza Sharifi

**Affiliations:** 1 Department of Genetics and Molecular Biology, School of Medicine, Isfahan University of Medical Sciences, Isfahan, Iran; 2 Department of Pathology and Laboratory Medicine, Tehran Heart Center, Tehran University of Medical Sciences, Tehran, Iran; 3 Department of Pharmacology and Toxicology, School of Pharmacy, Kerman University of Medical Sciences, Kerman, Iran; Gastroenterology and Hepatology Research Center, Afzalipour's Hospital, Imam Highway, Kerman, Iran; 4 Department of Cardiac Research, Tehran Heart Center, Tehran University of Medical Sciences, Tehran, Iran; Department of Clinical Pharmacy, School of Pharmacy, Tehran University of Medical Sciences; 5 Cellular and Molecular Research Center, Birjand University of Medical Sciences, Birjand, Iran

**Keywords:** bleeding, Warfarin, GATA4 gene, microRNAs, polymorphism

## Abstract

Bleeding is the most serious complication of warfarin anticoagulation therapy and is known to occur even at patients with therapeutic international normalized ratio (INR) range. Recently, it has been shown that microRNAs play a significant role in pharmacogenetics by regulating genes that are critical for drug function. Interaction between microRNAs and these target genes could be affected by single-nucleotide polymorphisms (SNPs) located in microRNA-binding sites. This study focused on 3′-untranslated region (3′-UTR) SNPs of the genes involved in the warfarin action and the occurrence of bleeding complications in an Iranian population receiving warfarin. A total of 526 patients under warfarin anticoagulation therapy with responding to the therapeutic dose and maintenance of the INR in the range of 2.0-3.5 in three consecutive blood tests were included in the study. Four selected 3'-UTR SNPs (rs12458, rs7294, rs1868774 and rs34669593 located in *GATA4*, *VKORC1*, *CALU* and *GGCX* genes, respectively) with the potential to disrupt/eliminate or enhance/create microRNA-binding site were genotyped using a simple PCR-based restriction fragment length polymorphism (PCR-RFLP) method. Patients with the rs12458 AT or TT genotypes of the *GATA4* gene had a lower risk of bleeding compared to patients with the AA genotype (adjusted OR: 0.478, 95% CI: 0.285-0.802, *P*= 0.005, OR: 0.416, 95% CI: 0.192-0.902, *P*= 0.026, respectively). 3'-UTR polymorphisms in other genes were not significantly associated with the risk of bleeding complications. In conclusion, the SNP rs12458A>T in the 3′UTR region of *GATA4* is associated with the incidence of warfarin-related bleeding at target range of INR, likely by altering microRNA binding and warfarin metabolism. Further genetics association studies are needed to validate these findings before they can be implemented in clinical settings.

## Introduction

Warfarin was introduced into clinical practice in the 1950s and since has become the mainstay of oral anticoagulant treatment in the world (Jones and Miller, 2011[18]). Over 60 years, warfarin has been in clinical use for treatment and/or prevention of pulmonary embolism, venous thrombosis, and other thromboembolic events associated with myocardial infarction, atrial fibrillation and the conditions requiring coagulation control (Wysowski et al., 2007[50]; Kimmel, 2008[20]). Despite approval of several new oral anticoagulant medications, warfarin is still prescribing as a beneficial anticoagulant in prosthetic heart valve (Anderson and Marrs, 2018[3]; Durães et al., 2018[9]) and in low socioeconomic populations (Desai et al., 2014[8]).

Considering the warfarin narrow therapeutic window, regular monitoring and maintaining a patient's international normalized ratio (INR) within a therapeutic range is demanded for achieving desired anticoagulation (Lee and Klein, 2013[22]; Emery, 2017[10]). INR is an international standardized number for the prothrombin time (PT). For most indications, the most widely accepted therapeutic INR range for warfarin treatment is 2.0 to 3.0 (Wadelius et al., 2009[48]; Lee and Klein, 2013[22]). However, in patients with valvular heart disease and those with prosthetic heart valves INR value increases to 3.5 (Goldsmith et al., 2002[13]).

Bleeding is the major adverse effect of oral anticoagulant therapy following the treatment and prevention of thromboembolic complications in usual care settings (Palareti et al., 1996[29]; Epstein et al., 2010[11]). Patients on warfarin are also at increased bleeding tendency (Campbell and Gallus, 2001[6]; Wu, 2018[49]). Warfarin-induced bleeding commonly occurs during the first 90 days of treatment, leads to increased morbidity, mortality, and higher cost of health care (Lund et al., 2012[23]; Nekkanti et al., 2012[28]). Typically, the incidence of bleeding is associated with the over-anticoagulation and increases parallel to an elevation of INR and fifty percent of bleeding events is observed when the INR value is less than 4.0 (Campbell and Gallus, 2001[6]; Lee and Klein, 2013[22]).

Although having INR within accepted therapeutic ranges reflects the optimal clinical outcomes of warfarin therapy, even when patients are within the therapeutic INR range can suffer serious bleeding complications (Pourgholi et al., 2016[30]). Several patient-related clinical factors including age, gender, concomitant drug intake, duration of warfarin use, concurrent diseases, history of bleeding complication, and warfarin indication have been considered to be risk factors for warfarin-related bleeding complications and also contribute to inter-individual variability in warfarin response (Jones and Miller, 2011[18]; Nekkanti et al., 2012[28]). Besides these patient and clinical factors, a major portion of the variation in warfarin response remains in part unexplained (Tang et al., 2017[45]). 

Accumulating evidence supports that genetics is a predictive factor of the final drug response and pharmacogenetics-based therapies with warfarin have been associated with a reduced risk of excessive anticoagulation and hemorrhage complications (Aithal et al., 1999[1]; Sridharan et al., 2016[41]). 

Products of two important genes,* VKORC1 *(vitamin K epoxide reductase complex subunit 1) and *CYP2C9*, are responsible for the majority of the pharmacodynamics and pharmacokinetics of warfarin, respectively (Farzamikia et al., 2018[12]; Wu, 2018[49]). Firstly, warfarin is metabolized by the hepatic CYP2C9 enzyme and then inhibits the coagulation cascade through the targeting of the VKORC1 enzyme to decrease the active vitamin K regeneration from vitamin K epoxide (Krajciova et al., 2014[21]; Sridharan et al., 2016[41]). 

There is overwhelming evidence that single nucleotide polymorphisms (SNPs) in the *CYP2C9* and *VKORC1* genes are strongly associated with variability in dose requirements and occurrence of bleeding complications during warfarin treatment (Jones and Miller, 2011[18]; Relling and Evans, 2015[34]). Various studies showed that *VKORC1* (−1639G>A) rs9923231 polymorphism may cause or be associated with up to forty percent of the dose variation in some populations and, in comparison with the wild type, individuals carrying this polymorphism showed lower warfarin requirements (Krajciova et al., 2014[21]; Bader and Elewa, 2016[4]). 

Numerous other genes including the microsomal epoxide hydroxylase (*EPHX1*), γ-glutamyl carboxylase (*GGCX*), calumenin (*CALU*) and GATA binding protein 4 (*GATA4*), also have been implicated to determine the inter-individual variability in warfarin response (Mwinyi et al., 2009[27]; Shahabi et al., 2018[39]). To our knowledge, few studies have directly investigated the effect of 3′-untranslated region (3′UTR) SNPs of warfarin-related genes on clinical outcomes such as the incidence of bleeding events due to warfarin therapy. In this study, we selected 4 SNPs with the potential to disrupt/eliminate or enhance/create miRNA-binding sites in the 3′UTR of some genes involved in warfarin action. The aim of this study was to explore the association between these variants and bleeding complications in Iranian patients receiving warfarin with an INR within the therapeutic range. 

## Methods

### Study design and study population

The present study, which was planned as cross-sectional, was conducted between January and September 2018, at Tehran Heart Center (THC). A total number of 526 patients with the following inclusion criteria were included the study comprising patients who used warfarin following atrial fibrillation, valvular disease, deep vein thrombosis (DVT), pulmonary thromboembolism (PTE) and the use of warfarin and response to the therapeutic dose with INR maintenance in the range of 2.0-3.5 in three consecutive blood tests. Patients were enrolled in the study when written informed consent was obtained from eligible patients. The patients with serious complications, including malignancies, acute infections, alcohol intake, using glucocorticoids and herbal drugs, pregnancy, and other concurrent treatment interacting with warfarin were excluded from this research. The study design was confirmed by the local Ethics and Human Rights Committee and carried out according to the guidelines of the Helsinki Declaration.

### Collection of clinical data

Data regarding the gender, age, warfarin therapy duration, body weight, valve prosthesis, height, daily warfarin dose, concurrent medication, indication for therapy, INR measurements, comorbidity, and history of bleeding complication were gathered from the medical records of patients. Other data including blood pressure, diet, medication histories, alcohol, and changes in warfarin dose were recorded at each physician visit. Then, we obtained variables concerning patient's characteristics such as laboratory information and cardiovascular risk factors by using hospital database. The incidence of different types of bleedings, including epistaxis, hematuria, gingival, gastrointestinal, vaginal, and subconjunctival was recorded throughout the entire period of observation and defined as those needing medical intervention to treat or stop bleeding. In the present study, warfarin therapy duration was defined as the number of days between beginning of warfarin administration and the last clinical visit.

### Laboratory analysis

Two blood samples were taken from each patient for INR measurement, and DNA extraction. About 5 ml of whole blood was collected in tubes containing ethylenediamine tetraacetic acid (EDTA) and frozen for DNA extraction and future use. For INR calculation, plasma was separated from the blood sample by centrifugation at 1500 rpm for 15 min and then the PT with INR were measured with a coagulation analyzer-the ACL-ELITE-PRO (Instrumentation Laboratory, USA).

### Single nucleotide polymorphism selection

Several specialized miRNA-target SNP databases (miRNASNP2, miRSNP, miRdSNP, MicroSNiPer) were used to identify SNPs with the potential to disrupt/eliminate or enhance/create miRNA-binding sites within the 3′UTR of genes involved in the action of warfarin. Among the suggested SNPs, those with MAF (minor allele frequency) higher than 0.1 were chosen. Moreover, minimum free energy of hybridization (MFE) for the selected 3′UTR SNPs were calculated using RNAcofold Web Server for the wild and the variant alleles (Gruber et al., 2008[14]). Finally, four polymorphisms in the 3′UTR of four genes involved in the warfarin action were selected. These SNPs included: rs1868774G>A, rs34669893G>A, rs12458A>T, rs7294G>A in the 3′UTR regions of *CALU*,* GGCX, GATA4*, and *VKORC1 *genes respectively. In silico predicted miRNAs relating to these SNPs also are listed in Table 1(Tab. 1). 

### DNA extraction and genotype analysis

Leukocyte genomic DNA of each patient was extracted from peripheral blood samples containing EDTA according to the 'salting out' method. DNA was quantified by measuring the optical density (OD) at λ = 260 nm. The 260/280 ratio was used to assess the quality of DNA being close to 1.8. The SNP genotypes of *VKORC1 *(rs7294), *CALU *(rs1868774), *GGCX *(rs34669893) and *GATA4 *(rs12458) variants, were determined by restriction enzyme digestion following polymerase chain reaction amplification of genomic DNA using designed primers and corresponding restriction enzymes listed in Table 2(Tab. 2). The restriction enzyme (1 U) was added to PCR products and digestion was performed. After incubation for overnight (16 h) at 55 °C, 37 °C and 60 °C for SmlI, AluI and MwoI enzymes, respectively, the digested fragments and products of PCR were analyzed on ethidium bromide-stained agarose gel and visualized under ultraviolet light. Finally, the genotypes of each sample were determined from the digestion patterns of bands in the gel electrophoresis. 

### Statistical analysis

In this study variables were evaluated for normal distribution by using the Kolmogorov-Smirnov normality test. The independent two-sample Student t-test (or Mann-Whitney U test if required) and the chi-square test were used to compare the difference between two groups of patients with bleeding complications (bleeding group) and without bleeding complications (non-bleeding group) for continuous variables and categorical variables, respectively. In both groups, Hardy Weinberg equilibrium for all polymorphisms was analyzed by Chi-square test. Association between the genotypes and bleeding following warfarin therapy was recomputed using multivariate logistic regression after adjustment for covariates. Odds ratio and 95% confidence interval (CI) were determined to measure the association between variables. A *p*-value ≤0.05 was declared statistically significant. All statistical calculations were performed by PASW Statistics for Windows, Version 18.0 (SPSS Inc., Chicago, USA).

## Results

Of a total of 526 patients meeting the eligibility criteria, 82 (15.6 %) patients had bleeding complications with a mean INR of 2.7 ± 0.4 that was in the therapeutic range. Eighty-three bleeding events (minimal hemorrhage) were observed and one patient had 2 bleeding episode (epistaxis and gingival). Major life-threatening bleeding complications were not observed among patients. The most frequent site of bleeding was epistaxis 53 % (44/83). The other sites of bleeding arranged in descending order of frequency included; Hematuria and gingival (each was 11 %), gastrointestinal (10 %), vaginal (4 %) and subconjunctival hemorrhage (3 %). Patients had a mean age of 58 ± 12 years (between the ages of 16 and 88 years), 47 % were male and 53 % female. Mean stable warfarin dose was 36 ± 17 milligram per week and the mean length of warfarin therapy was 1446 ± 790 (133-4432) days. Indications for warfarin therapy were included prosthetic valve (63 %), atrial fibrillation (21 %), thromboembolic disease (12 %) and other indications (4 %).

Demographic characteristics of study participants are demonstrated in Table 3(Tab. 3). Data showed significant differences in the age, INR, antibiotics and CYP inducers between bleeding and non-bleeding groups. There was no significant difference in gender, comorbidities, average warfarin dose, and body mass index between bleeding and non-bleeding groups.

The distribution of all four SNPs was found to be in Hardy Weinberg Equilibrium (HWE χ2<3.84 and p>0.05). Table 4(Tab. 4) provides the genotype frequencies for the analyzed polymorphisms and the logistic regression model before and after adjustment that *GATA4* and *GGCX* adjusted for age and CYP inducers, *VKORC1* and *CALU* adjusted for age. Results showed that patients with heterozygous (AT) and homozygous mutant (TT) genotypes of* GATA4* rs12458, had a lower risk of bleeding than patients with the homozygous wild (AA) genotype (adjusted OR: 0.478, 95% CI: 0.285-0.802, *P*= 0.005, OR: 0.416, 95% CI: 0.192-0.902,* P*= 0.026 respectively). There were no significant associations between other selected variants in *VKORC1*, *CALU*, and *GGCX *genes and the risk of bleeding events.

## Discussion

The current study found that *GATA4* rs12458 genotype is significantly associated with bleeding complications at normal INR in Iranian patients during warfarin treatment. As far as we know, this is the first attempt to empirically examine the relevance of genetic variations of *GATA4* and *CALU* and *GGCX* in bleeding complications despite of normal INR.

*GATA4*, a member of transcription factor family GATA containing two zinc finger DNA binding domains, consists of 442-amino acids and binds to a (A/T)GATA(A/G) sequence (Reamon-Buettner et al., 2007[33]). *GATA4* is expressed in several endoderm- and mesoderm-derived tissues such as liver, small intestine, heart, lungs, and gonads as a critical tissue-or cell type-specific gene regulator (Molkentin, 2000[26]). When expressed in the embryonic heart and yolk sac endoderm, *GATA4* is involved in cardiac development and cardiomyocyte differentiation in the embryonic and postnatal heart (Pulignani et al., 2016[31]). *GATA4* also has been shown to specifically regulate expression of the interleukin-5 gene in a human T-cell line (Molkentin 2000[26]). In humans, *GATA4* gene mutations are highly associated with congenital heart defects (Reamon-Buettner et al., 2007[33]). 

Moreover, it has previously been shown that *GATA4*, as a liver-specific transcription factor, regulates the expression of different liver detoxifying enzymes and transporters (Mwinyi et al., 2009[27]; Jeong et al., 2015[17]). To date, several studies have shown that* GATA4* is strongly involved in the transcriptional activation of human CYP2C9, a principal metabolizing enzyme in the warfarin pharmacological pathway (Mwinyi et al., 2009[27]; Van Schie et al., 2012[47]). There are some essential regulatory elements such as several GATA4-binding motifs in the proximal promoter region of the *CYP2C9* gene enabling *GATA4* to affect the expression of the CYP2C9 enzyme (Jeong et al., 2015[17]). 

Mwinyi et al. (2009[27]) reported upregulation of promoter activity in wild-type *CYP2C9* by liver-specific transcription factor *GATA4*. They investigated four putative GATA binding sites within *CYP2C9* promoter sequence using luciferase gene reporter and electrophoretic mobility shift assays. Interestingly, mutations located in GATA4-binding sites dramatically decreased this induction (Mwinyi et al., 2009[27]). A recent investigation described that polymorphisms in nuclear receptor genes are potentially associated with alteration in *CYP2C9* expression and altered drug metabolism (Shahabi et al., 2018[39]). Genetics variations in *GATA4* have been postulated to play a significant role in the inter-individual variability in drug response (Van Schie et al., 2012[47]). Results of a research carried out on a population of Korean patients with prosthetic cardiac valve showed an association between *GATA4* variants rs2645400 and rs4841588 and warfarin dose requirements through the *CYP2C9* expression regulation (Jeong et al., 2015[17]). Another study conducted on a population of Netherlands patients revealed that one variant in *GATA4* influenced the dose requirement of a warfarin derivative, acenocoumarol, in the patients having a specific *CYP2C9* genotype (Van Schie et al., 2012[47]). 

The relationship between warfarin-induced bleeding and *CYP2C9* genotypes has been widely investigated (Sanderson et al., 2005[37]; Samardžija et al., 2008[36]; Ucar et al., 2013[46]; Yang et al., 2013[51]; Pourgholi et al., 2016[30]). To examine this issue, one group carried out an experiment on 185 patients who were under anticoagulation therapy with warfarin to find if* CYP2C9* gene variants can affect the occurrence of bleeding complications. They observed a significantly higher rate of bleeding complications in CYP2C9*2 and *3 carriers compared to non-carriers (Margaglione et al., 2000[24]). Results of a cohort study on two hundred patients showed that CYP2C9*2 and *3 polymorphisms are associated with over-anticoagulation and incidence of bleeding during warfarin therapy (Higashi et al., 2002[16]). Kawai et al. identified that patients with a CYP2C9*3 polymorphism had a significantly increased risk of bleeding after receiving warfarin (Kawai et al., 2014[19]). Moreover, results of a meta-analysis and systematic review from 22 reports indicated that CYP2C9*2 and CYP2C9*3 genotypes are genetic risk factors for warfarin hemorrhagic complications (Yang et al., 2013[51]). In contrast, findings of a study conducted on Korean population with mechanical cardiac valves showed that *CYP2C9 *rs1057910 and *VKORC1* rs9934438 variants were not significantly associated with increased risk of bleeding due to warfarin therapy at therapeutic INR (An et al., 2014[2]). In a recent study, Sridharan et al. confirmed findings of previous studies regarding the significant association between *CYP2C9* genotypes and increased risk of bleeding events following warfarin therapy (Sridharan et al., 2016[41]). In a cross-sectional study on 552 warfarin-treated patients with target INR level of 2.0-3.5, Pourgholi et al. demonstrated that SNP C609T within quinoneoxidoreductase 1 (*NQO1*) and haplotypes of *CYP2C9* (1*2 or 1*3) significantly associated with bleeding complications (Pourgholi et al., 2016[30]). In view of all that has been mentioned so far, one may suppose that significant association between *GATA4* SNP and bleeding events, observed in the present study, may be due to the transcriptional effect of *GATA4* on *CYP2C9* gene.

Previous investigations have demonstrated that variants of *VKORC1* are associated with bleeding risk upon warfarin therapy (Schwarz et al., 2008[38]). *VKORC1* (1639G>A) functional promoter SNP showed an increased incidence in occurrence of bleeding complications during warfarin therapy in AA genotype group (Lund et al., 2012[23]). In a study, Mazur-Bialy et al. reported that CYP2C9*3/ VKORC1*2A haplotype in a Polish patient had a higher sensitivity and bleeding event in warfarin-treated patients (Mazur-Bialy et al., 2013[25]). Sridharan et al. have also shown that *VKORC1* (1639G>A) mutant allele is associated with increased bleeding tendency due to warfarin therapy (Sridharan et al., 2016[41]). Biswas et al. have a recent publication resulted from a study on a population of Indian patients in which they showed that AG/AA haplotype of *VKORC1 *(1639G>A) rs9923231 was associated with increased risk of warfarin bleeding episodes (Biswas et al., 2018[5]). In contrast to these findings, in our investigation, no evidence of an association between selected SNP in 3'-UTR of *VKORC1* and occurrence of bleeding was detected. Moreover, in the present study, no association was observed between selected SNPs in *CALU* and *GGCX* genes and bleeding episodes.

MicroRNAs, an abundant group of single-stranded noncoding RNAs comprised of 18-24 nucleotides, binding to the 3'-UTR sequences of target mRNAs and leading to degradation or translation inhibition of their target genes, play significant regulatory roles in the post-transcriptional level (Ciccacci et al., 2015[7]; Rad et al., 2018[32]). Several reports have shown that SNPs within microRNA binding sites could affect the expression of target genes and influence on drug responses or risk of a disease (Swart and Dandara, 2014[42]; Pulignani et al., 2016[31]). Shomron et al. identified two binding sites for miR-133 and miR-137 on *VKORC1* mRNA and suggested regulation of *VKORC1* expression by these miRNAs (Shomron, 2010[40]). This result is in accord with the finding of a recent investigation indicating that miR-133a interacted with the 3′UTR of *VKORC1*. It is noticeable that focus of this study was on the SNPs of *mir-133* gene, *MIR133A2* and *MIR133B*, which encode miR-133a-2 and miR-133b, respectively, to examine the association between *miR-133* SNPs and stable warfarin dose in Han Chinese patients with mechanical heart valve replacement (Tang et al., 2017[45]). Results of an experiment carried out by *in vivo, in vitro, *and* in silico* analyses, demonstrated that suppression of *CYP2C9* expression correlated with the expression of miR-128 in hepatocellular carcinoma tumor tissues (Yu et al., 2015[52]). 

In our study, we identified six miRNAs which their binding site disrupt/enhance on the *GATA4* mRNA following rs12458A>T SNP. These miRNAs included; miR-556-5p, miR-4279, miR-500b, miR-502-5p, miR-526b, and miR-362-5p. So far, the association between these miRNAs, probably target the 3′UTR of *GATA4*, with bleeding complications after warfarin therapy have not been reported in the literature. Two independent studies reported that SNPs in the 3'-UTR of *GATA4* gene may play a role in the pathogenesis of congenital heart disease (CHD) probably by modifying miRNA post-transcriptional gene regulation (Reamon-Buettner et al., 2007[33]; Pulignani et al., 2016[31]). Another study also points out that common variants in the 3′UTR of *GATA4* are involved in congenital heart defects (Sabina et al., 2013[35]). Han et al. investigated the GATA4-regulating transcriptional vs. post-transcriptional mechanisms in the heart and reported an increased GATA4 protein level during cardiac hypertrophy resulted possibly from miR-26b post-transcriptional regulation (Han et al., 2012[15]). It has been shown that a transcription factor named pregnane X receptor (PXR), involved in expression regulation of drug-metabolizing enzymes comprising cytochrome P450 3A4 (CYP3A4), post-transcriptionally is regulated by miR-148a influencing the levels of CYP3A4 in human liver (Takagi et al., 2008[44]). Interestingly, in a study Takagi et al. found that one another transcription factor, HNF4A (hepatocyte nuclear factor 4 alpha) which regulates expression of transporters and endo/xenobiotic-metabolizing enzymes, was down-regulated by miR-24 and miR-34a thus decreased cytochrome P450 7A1 and 8B1 genes in HepG2 cells (Takagi et al., 2010[43]). According to these study reports, our results can be explained by considering that six identified miRNAs could possibly modify the activity of the transcription factor* GATA4* following rs12458 polymorphism and, as mentioned above, the *GATA4* is a modulator of *CYP2C9* expression, therefore, it is considerable that, compared with the wild-type allele, heterozygous and homozygous mutant allele of rs12458A>T could disrupt or enhance miRNA-binding sites within the 3′UTR of *GATA4* gene resulting in altered warfarin metabolism that may lead to bleeding complications.

A key strength of the present study is that we performed this study on a group of Iranian patients under warfarin therapy aiming for an optimal INR level of 2.0 to 3.5 who continued the INR range during three consecutive blood tests. This was of a great advantage to investigate possible consequences of specific genetic variants related to warfarin on the risk of bleeding complications at therapeutic range of INR after adjustment for multiple demographic and clinical factors. Moreover, this study suffer from some limitations require to be considered. This research is limited by its cross-sectional design. Moreover, the limited number of patients with bleeding led to insufficient statistical power. Another limitation is that we do not examine all of the polymorphisms with the highest amount of total free energy. However, we might lose some of the critical variants and their relevant miRNAs. On the other hand, the functional effect of bleeding-associated SNP on target interaction of six suggested miRNAs was not investigated. Finally, this study was conducted in a single center and therefore findings cannot be extended to other ethnic groups and further studies need to be carried out on populations of different genetic backgrounds.

## Conclusion

The results of this investigation show that SNP rs12458 within a specific region of *GATA4* 3′UTR is associated with bleeding complications of warfarin at therapeutic range of INR, likely by altering the transcriptional gene regulation *CYP2C9*. Further genetics association studies are needed to examine the effects of different variants in the 3′UTR region of *GATA4* gene on the occurrence of bleeding events in patients under anticoagulant therapy and interpret and generalize these results before they can be implemented in the context of real clinical practice. Moreover, more functional studies on the influence of different SNPs in miRNA binding sites will be a base line to better give further insights into the mechanisms of action of drug or chemical responses and individualized therapy in advanced.

## Funding

This study was funded by Isfahan University of Medical Sciences [grant Number: 396319].

## Conflict of interest

The authors declare that they have no conflict of interest.

## Ethical approval

This study was approved by the local ethics committee of Isfahan University of Medical Sciences, IRAN. The studies have been approved by the appropriate institutional and/or a national research ethics committee and have been performed, in accordance with the ethical standards, as laid down in the 1964 Declaration of Helsinki and its later amendments or comparable ethical standards.

## Informed consent

Informed consent was obtained from all individual participants included in the study.

## Figures and Tables

**Table 1:**
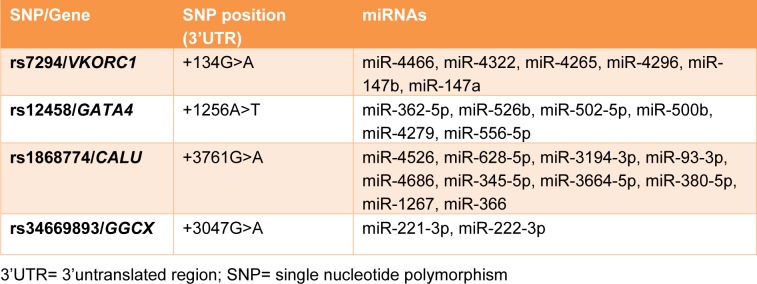
Selected SNPs and their relevant genes and miRNAs

**Table 2:**
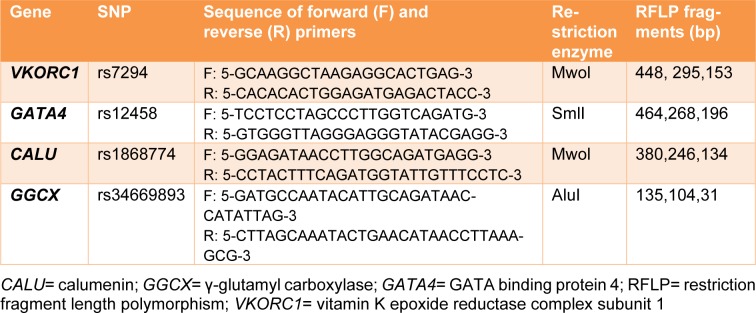
Primer sequences and RFLP fragments of suggested genes

**Table 3:**
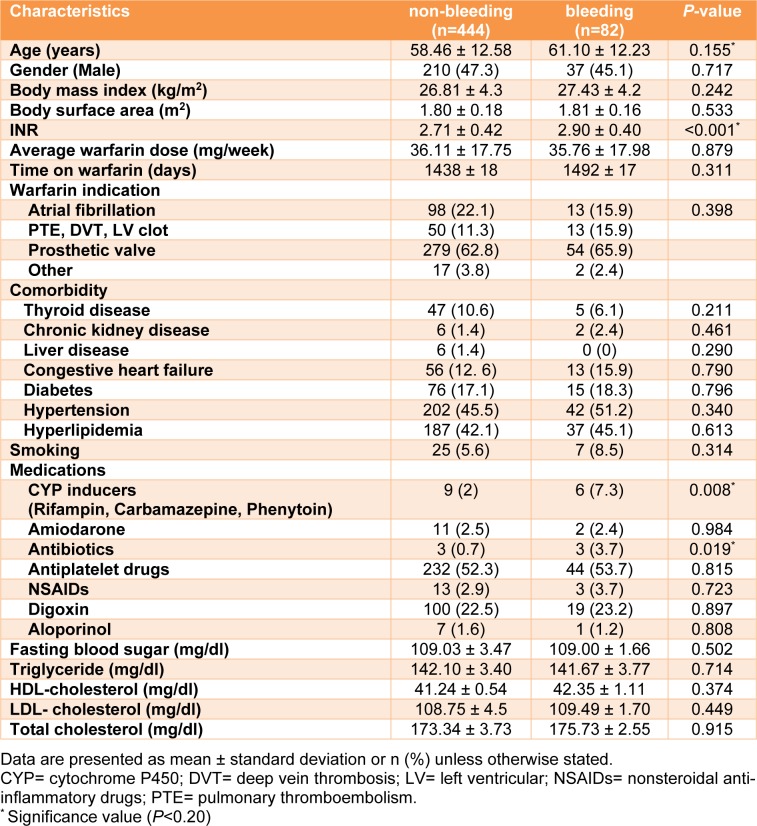
Demographic, clinical and laboratory characteristics of the study groups

**Table 4:**
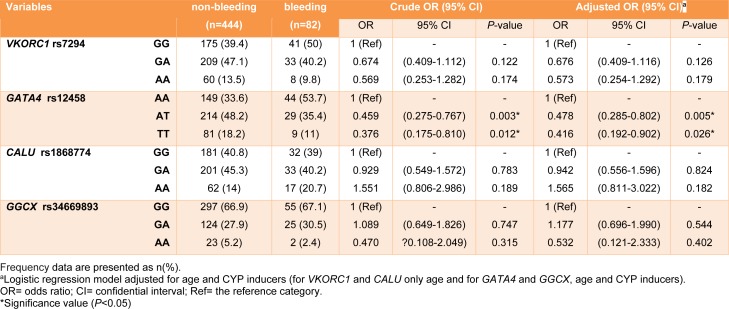
Genotype frequencies of examined polymorphisms and logistic regression analysis showing odds ratios for bleeding complications
